# The effect of the emotion regulation training on the resilience of caregivers of patients with schizophrenia: a parallel randomized controlled trial

**DOI:** 10.1186/s40359-021-00542-5

**Published:** 2021-03-02

**Authors:** Maryam Behrouian, Tahereh Ramezani, Mahlagha Dehghan, Abdoreza Sabahi, Batool Ebrahimnejad Zarandi

**Affiliations:** 1 Yazd University of Medical Sciences, Yazd, Iran; 2 Department of Public Health, Faculty of Nursing and Midwifery, Kerman University of Medical Sciences, Kerman, Iran; 3 Nursing Research Center, Kerman University of Medical Sciences, Kerman, Iran; 4 Department of Critical Care Nursing, Razi Faculty of Nursing and Midwifery, Kerman University of Medical Sciences, Haft-Bagh Highway, Kerman, Iran; 5 Kerman University of Medical Sciences, Kerman, Iran; 6 Shaheed Beheshti Medical Center, Kerman University of Medical Sciences, Kerman, Iran

**Keywords:** Emotion regulation, Resilience, Caregiver, Schizophrenia, Cognitive-emotional training

## Abstract

**Background:**

Schizophrenia is the most severe mental chronic disabling disease that the majority of the patients need constant care in a variety of aspects. Regarding the role of family caregivers in taking care of these patients, caregivers need to be resilient, in addition to other psychological traits, to adapt to the circumstance. This study aimed to investigate the effect of the emotion regulation training on the resilience of caregivers of patients with schizophrenia in southeastern Iran.

**Methods:**

The study was a parallel randomized controlled trial. Seventy caregivers of patients with schizophrenia were selected by convenience sampling method and randomly assigned to an emotion regulation training group and a control group. The intervention group received eight 90-min training sessions (one session weekly) about emotion regulation. The participants completed the Conner–Davidson resilience scale before and one month after the intervention.

**Results:**

The mean scores of the resilience increased in the control and intervention groups at the end of the study. A significant difference was found between the two groups (p < 0.001). At the beginning of the study, the mean score of the resilience was 59.94 in the control group and 51.97 in the intervention group. However, the mean score of the resilience in the control group was 61.28 after the intervention, which was not significant, but it was 69.08 in the intervention group, which was significant. A significant difference was observed between two groups in the mean scores (p = 0.01).

**Conclusions:**

According to the results of this study, cognitive and metacognitive skills of emotion regulation can be suggested as one of the methods for increasing the psychological well-being of schizophrenia patients’ caregivers. The increase of mental well-being and resilience of caregivers can help them better manage a patient with schizophrenia.

*Trial registration* IRCT registration number: IRCT2017061733997N2, Registration date: 2017-08-16, 1396/05/25, Registration timing: prospective, https://en.irct.ir/trial/26116

## Background

Schizophrenia is the most severe chronic disabling disease, which is associated with impaired social and occupational abilities. Even though a small percentage of people in the community suffer from the disease, it has enormous societal consequences. The functioning of these patients is impeded in various occupational, educational, social, and interpersonal, and self-care aspects, and they need permanent care [1]. Families are the primary caregivers of the patients [2] and face unpredictable stressors and strange behaviors of the patient with schizophrenia. Therefore, in addition to primary care, they should adapt to and manage the patient's symptoms [3].

A review of the literature suggests that the diversity and great responsibility of care roles impair the resilience process of the caregivers, who requires it to resist and endure the conditions of the patient with schizophrenia [4]. In the literature, the term resilience ranges from prevention of mental health disturbance to successful adaptation and quick recovery after experiencing life adversities and may include posttraumatic psychological growth. Three important psychological building blocks of resilience are experiencing positive emotions, secure attachment, and having a purpose in life [5]. There is a positive relationship between resilience and mental health [6]. Caregivers, who are more resilient have higher mental health and perform well in dealing with a variety of emotional, cognitive, behavioral, and social problems [7]. In addition, resilient families take care of patients with schizophrenia better [8].

Some therapeutic interventions, including training, support, and psychotherapy could have a dramatic effect on reducing negative emotional excitement of the family caregivers and may predispose to improving the quality of care and physical and mental health of caregivers [9]. Emotion regulation helps people have a grasp of their emotions. It is a process of how any individual experiences and expresses his/her emotions [10]. All strategies used to reduce, increase, or maintain positive or negative emotions are referred to as emotion regulation [11]. Furthermore, being able to regulate emotions is associated with high levels of resilience [12].

Since most discharged patients suffer from chronic mental illness, taking care of them in the family without enough information or skills is difficult, and training the patient and family should be the top priority of community-based health programs in the world [13]. Chang et al. (2016), Rodríguez-Sánchz et al. (2011), and Tremont et al. (2014) concluded that families spent most of their time taking care of psychiatric patients and did not have any time for their leisure time. Therefore, chronic illness can lead to a poor family functioning. In addition, caregivers do not have a comprehensive perception of different aspects patients care needs such as communication, roles, emotional response, and overall performance. Therefore, they may experience negative life events while caring patients with schizophrenia, which lead to development of depression, anxiety and stress in caregivers [14–19].

Regarding the search of the available literature and to the best of authors’ knowledge, there was no program aimed to provide psychological support and social/psychological skills training to family members of people affected with schizophrenia in the Iranian health system. Therefore, the aim of this study was to determine the effect of the emotion regulation training on the resilience of caregivers of patients with schizophrenia.

## Methods

The study adheres to CONSORT guidelines.

### Study design and setting

This study was a parallel randomized controlled trial. The participants were family caregivers of patients with schizophrenia referred to the psychiatric wards in 2016–2017 in Kerman, Iran.

### Sample size and sampling procedure

According to the previous study [19, 20], (post intervention stress score in intervention and control groups respectively: μ1 = 13.4, μ2 = 10.8, S1 = 3.89, S2 = 3.48), 32 participants were estimated for each group with a confidence interval of 95% and type II error of 20%. According to the probability of dropouts, 35 samples were selected in each group. Eligible family caregivers were selected by convenience sampling method and randomly assigned to each group using block randomization method. A label of A or B (A = intervention, B = Control) was assigned to each group (block size = 4). We used free online software (https://www.sealedenvelope.com/simple-randomiser/v1/lists) to generate the randomization list [21]. The third author generated the random allocation sequence, and the first author enrolled participants and assigned participants to the groups.

### Instruments

The first questionnaire was about the demographic data of patients and caregivers. Patient's socio-demographic characteristics included age, sex, level of education, duration of illness, marital status, occupation, economic status, insurance status, living place, housing situation, and the number of family members (sister, brother, daughter, son, and spouse). Caregiver's socio-demographic characteristics included age, gender, educational level, relation to the patient, duration of care, occupational status, economic status, insurance status, living place, housing situation, the number of family members (sister, brother, daughter, son, and spouse), and the presence of another person with a mental health condition in the family.

The Conner–Davidson resilience scale (CD-RISC) [22] is used to measure the level of resilience. This questionnaire has 25 items on a five-point Likert scale (0–4). The total scores range from zero to 100. The cut-off point for this questionnaire is 50, with higher score indicating higher resilience. The authors of the CD-RISK do not advise the use of other scores i.e. scores of CD-RISC subscales. The internal consistency (Cronbach’s α) for the full scale was 0.89. Test–retest reliability was assessed in 24 subjects from the clinical trials of generalized anxiety disorder and posttraumatic stress disorder groups. The mean (SD) scores at time 1 [52.7 (17.9)] and time 2 [52.8 (19.9)] demonstrated a high level of agreement, with an intraclass correlation coefficient of 0.87. For Convergent validity, CD-RISC scores were positively correlated with the Kobasa hardiness measure in psychiatric outpatients (Pearson r = 0.83, p < 0.0001). Compared with the Perceived Stress Scale (PSS-10), the CD-RISC showed a significant negative correlation (Pearson r = 0.76, p < 0.001) [22]. Jowkar in Iran reported the scale reliability of 0.93 by using the Cronbach's alpha [23]. Ahangarzadeh Rezaei and Rsoli checked the psychometrics properties of the scale in adolescents with cancer. They reported the content validity of the scale as SCVI = 0.98, and test–retest reliability was assessed with Pearson correlation coefficient (r = 0.404, p < 0.05) [24]. In addition, the Cronbach's alpha of this scale in the current study (before intervention) was 0.91.

### Data collection and analysis

Sampling started after the necessary permissions were obtained from the authorities of the psychiatric clinics in Kerman. Primarily, the individual characteristics of patients with schizophrenia were checked and recorded. Patients with schizophrenia, who were admitted to the psychiatric center more than once were selected by a psychiatrist. Then, the family member who was responsible for hygiene, treatment, care, and support of the patient was invited to participate in the study. The inclusion criteria were age over 15 years old, being able to communicate, no history of participation in similar training sessions, and no history of severe physical illnesses and psychiatric disorders. The written informed consent form was obtained after the study method was explained. Caregivers, who were absent in more than two training sessions were excluded. Samples in the control group received the routine care, so psychiatric nurses and psychologists gave them advice if necessary. The families were offered to refer to psychologist offices and centers in case of any problem.

The samples of intervention group were divided into groups of five to six based on conditions such as distance and working conditions. Regarding the cognitive and metacognitive methods, a qualified clinical psychologist trained the emotion regulation to each group in eight 90-min sessions (one session per week) in a quiet situation in the hospital. The training was carried out through lecture, questioning and answering method, and booklet. The content of the training in each session was as follows: 1st session: communicating and conceptualizing the necessity of using the emotion regulation training. Second session: training the awareness of positive emotions and their types. Third session: training the awareness of negative emotions and their types. Fourth session: traning the acceptance of positive emotions. Fifth session: traning the acceptance of negative emotions. Sixth session: training of the reappraisal and proper expression of positive emotions. Seventh session: training reassessment and proper expression of negative emotions. Eighth session: summarizing the traning and question and answer sessions. The training sessions were based on prior studies [19, 25]. The psychologist did the training based on ‘*the Dialectical Behavior Therapy Skills Workbook’* [26]*.* The given treatment was based on cognitive behavioral therapy principles. It is noteworthy that emotion regulation training aimed to reflect mechanisms of perseverative negative thinking and the emotional concomitants in response to stressors [27]. The emotion regulation training attempts to communicate with thoughts that avoid resistance or perceptual analysis rather than to test intrusive thoughts and ineffective beliefs. Therefore, this method can remove unsuitable thinking strategies about anxiety as well as inflexible monitoring of the threat [19, 28]. CD-RISC was collected from all the samples before and one month after the intervention.

To analyze the data, we used SPSS software version 25. Descriptive statistics were used to describe the characteristics of the samples (frequency, percentage, mean and standard deviation). Chi-square test, independent t-test, and Mann–Whitney U test were used to determine the similarity (differences) of the two groups regarding demographic variables. If parametric conditions (normal distribution and equality of variances) were met, we used parametric statistical tests (Independent t test for comparison between the intervention and control groups, paired t-test for comparing groups before and after the intervention). Otherwise, non-parametric tests (Mann–Whitney and Wilcoxon tests) were used. In addition, Chi-Square and McNemar's tests were used to compare the resilience intensity between two groups. The multivariate linear regression test was used to control the effect of the confounding variables.

## Results

The mean ages of caregivers in the control and intervention groups were 45.34 ± 12.63 and 46.68 ± 15.51, respectively. The majority of participants in both groups were female. The duration of care in the majority of cases was less than five years (control = 62.9% and intervention = 42.9%). The majority of the subjects (60%) in both groups had degrees higher than the diploma (upper secondary education). 40% and 54.3% of the participants in the control and the intervention groups were unemployed, respectively. The majority of the subjects in both groups were married (control = 65.7% and intervention = 68.6%). 37.1% of the samples in the control and 51.4% of the samples in intervention group had monthly income less than 500,000 IRR which was less than the national average. The majority of subjects in two groups had health insurance (control = 85.7% and intervention = 82.9%). Regarding these variables, there was no significant difference between the control and intervention groups. Furthermore, there was no significant difference between the two groups in living place, housing situation, number of family members (p > 0.05). 14.3% of the participants in the control group and 40% of the participants in the intervention group took care of a patient with another mental illness in the family, and there was a significant difference between the two groups in this variable (χ2 = 5.85, p = 0.02). There were no significant differences between the two groups in the variables related to the patient with schizophrenia (p > 0.05), except for the patient's sex (p = 0.002).

At the beginning of the study, the mean resilience scores in the control and intervention groups were 59.94 and 51.97, respectively. Therefore, there was a significant difference between the two groups (t = 2.34, p = 0.02) so that the resilience score of the intervention group was less than that of the control group. The mean resilience scores in the control group was 61.28 and in the intervention group was 69.08 after the intervention, and there was a significant difference between two groups (t = − 2.56, p = 0.01). The resilience score in the control group increased 1.34 points after the study, while the score of resilience increased 17.11 points the intervention group (Z = − 5.41, p < 0.001) (Table 1). In addition, the multivariate linear regression was used to check the confounding effects of the patient's gender and the presence of another person with a mental health condition in the family. The results showed that only the type of groups was related to the resilience score (B = 7.18; p = 0.02).

**Table 1:** Comparison of the mean of the resilience scores between the two groups

Group	Time							
	Before the Intervention		After the Intervention		Paired T-test	p Value	Within group differences	
	Mean	SD	Mean	SD			Mean	SD
Control	59.94	13.87	61.28	12.34	1.57	0.120	1.34	5.05
Intervention	51.97	14.66	69.08	13.18	8.33	0.001	17.11	12.15
Statistical Test	t $=\hspace{0.17em}$2.34		t $=\hspace{0.17em}$−2.56				Z $=\hspace{0.17em}\hspace{0.17em}$− 5.41	
p Value	0.020		0.010				< 0.001	
Effect size	0.56		0.61				1.69	

t = Independent t test, Z = Mann Whitney U test

According to the cut point of 50, 82.9% of the participants in the control and 42.9% of the participants in the intervention group were resilient at the beginning of the study. The percent of the control group did not change after the intervention, but it reached to 94.3% in the intervention group. There was a significant difference between the two groups in the resilience score at the beginning of the study, so that the frequency of resilient subjects in the control group was greater than that of the intervention group. There was no significant difference between the two groups after the intervention (Table 2).

**Table 2:** Comparison of the number of resilient individuals between the two groups

Time	Group					
	Control		Intervention		χ^2^ Test	p Value
	Frequency	Percentage	Frequency	Percentage		
Before the intervention						
Not resilient	6	17.1	20	57.1	11.99	0.001
Resilient	29	82.9	15	42.9		
After the intervention						
Not resilient	6	17.1	2	5.7	2.26	0.13
Resilient	29	82.9	33	94.3		
McNemar's tests p value	–	< 0.001				

## Discussion

The aim of the present study was to determine the effect of the emotion regulation training on the resilience of caregivers of patients with schizophrenia. The results of this study showed that the resilience scores of the control and intervention groups had 1.34-point and 17.11-point increases, respectively before the intervention compared with after the intervention. 82.9% of the control group and 94.3% of the intervention group were resilient at the end of the study. In other words, the increased resilience in the intervention group was significantly better than that of the control group.

Min et al. [29] studied cognitive emotion regulation strategies contributing to resilience in patients with depression and/or anxiety disorders and indicated a significant correlation between cognitive emotion regulation strategies and resilience. Their results are in line with the result of this study in which emotion regulation is positively effective in increasing resilience. Training strategies for managing stress and anxiety are among the factors in this study that improved the level of resilience. Pejičić er al. [30] found that the mean resilience score was 80.4 ± 12.8 in general population group, which is far more than the mean resilience score of the family caregivers of patients with schizophrenia in this study. These results indicate the need for training programs like emotion regulation for schizophrenia patients’ caregivers. Vaughan et al. [31] examined 277 patients with cancer and showed that the effect of resilience on distress was fully mediated by emotion regulation, which is consistent with the results of this study. Steinhardt and Dolbier [32] showed that individual resilience could be enhanced by training, which might have a positive effect on significant clinical outcomes. Therefore, training emotional regulation refers to a useful and practical intervention to promote well-being, individual satisfaction and resilience [19, 33].

Improving resilience can improve the adaptation by managing stress and anxiety in cognitive and metacognitive aspects [19]. The training increases positive aspects of cognitive-emotional regulation, including positive re-focusing (thinking about a happy subject instead of thinking about an actual incident), re-focusing on planning (thinking about steps to overcome an adverse event, or changing it), positive reassessment (thinking about the positive aspects of an event or personal promotion), adoption of a perspective (thinking that the event is less important than other events), and improvement of subjects’ problem-solving ability [34].

Contrary to the results of the present study, the findings of some studies showed that the training was ineffective for resilience improvement. In this regard, we can mention the studies by Luthar et al. and Williams and Bryan [35, 36]. Given the inconsistent results of the studies mentioned above, there might be some weaknesses in cognitive and metacognitive training in coping with problems or the economic, environmental, sociocultural problems in the studied communities were the factors affecting the inconsistency with the results of this research.

Discussion with participants showed that the process helped them enjoy their lives, identify their destructive ideas and feelings, and try to change those feelings, do not give up, interpret their ideas and treat them appropriately. In addition, the training of effective interpersonal communication led participants to solve their problems rather than having mental rumination and blaming themselves and others, have more confidence in dealing with others and the patient, and have the ability to cope effectively and logically with irrational demands. Therefore, a person who is capable of solving a high-level problem can restrain his impulses well, gain the ability to return to his position after misfortunes,successfully adapt to them, increase his sense of independence and face life events better and more independently [37].

The study reported here aimed to help the participants manage their emotions and give meaning to their lives, escape from the vague conditions they thought they had. It also helped them not get tired of patient care, not consider themselves to be influenced by external or environmental activities, and act as an active and selective person, and thereby reduce the sense of helplessness. As nurses are the one of the main healthcare providers of patients with schizophrenia and their families, they should strengthen and develop resilience skills to deal with difficulties, adapt to new conditions, have realistic and positive expectations, and provide a better care for their patients and client. Studies have shown that people with high resilience are less prone to stress disorders and are more likely to remain healthy [38]. The present study can also help nurse leaders understand how to develop the interventions, which may improve nurses’ skills about using resilience training for patients with schizophrenia and their families. In addition, the present study can also encourage the healthcare policy makers to use such psychological interventions for medical students particularly nursing students, in which psychosocial needs were superior to other needs [39, 40]. Although there were some similar studies, which examined the effect of emotion regulation training/therapy on caregivers of patients with mental disorders [19, 41, 42], the present study seems to be among the first studies on the caregivers of patients with schizophrenia. Concerning the high level of psychological disorders, including stress, anxiety, and depression and resilience deficiency in this population [19], it is essential to provide efficient programs and interventions such as emotion regulation training/therapy to alleviate their problems. However, further studies are recommended to support the results of the present study.

One of the limitations of this study was that the samples were selected only from the psychiatric departments of one city and the schizophrenia patients’ caregivers, so the results should be generalized to other populations with caution. Restricting the study to the main caregiver of a patient with schizophrenia and not paying attention to other family members of patients is another limitation of the present study. Furthermore, not including the type of schizophrenia that the patients suffered form is another limitation. Assessing emotion regulations performance of the caregivers before and after the study could give much useful information, which was not considered in this study. In addition, our study was poorly controlled. The fact that the control group was a no-treatment group, without any placebo-treatment, should be mentioned, and it cannot be excluded that nonspecific attention for their coping problems might have influenced the positive results of the experimental group. Finally, we asked the caregivers if they had a patient with another mental illness in the family and we did not directly ask them if they were their main caregiver as well. This issue may be one of the reason that the participants in intervention group were less resilient at the beginning of the study compared with the control. However, we tried to check the confounding effect of this variable using multivariate linear regression.

## Conclusions

The results of this study showed that focus of training on emotional regulation as cognitive and metacognitive training significantly increased the resilience of schizophrenia patients’ caregivers. Therefore, the appropriate implementation of this therapeutic approach is one of the effective methods for improving resilience and is suitable for use in medical centers. Regarding the continuous and long-term care of patients with mental disorders, which is likely to reduce resilience and compatibility in caregivers, it is necessary to pay attention to the quality and content of the provided training. We can increase caregivers' compatibility by training them how to manage stress, anxiety, and depression.

## Data Availability

The datasets used for the current study are available from the corresponding author upon request.

## Abbreviations

CD-RISC: The Conner–Davidson Resilience Scale
SD: Standard Deviation
PSS: Perceived Stress Scale
ANOVA: Analysis of Variance
ANCOVA: Analysis of Covariance
